# “Name it to tame it”: piloting a narrative medicine clinical intervention for adolescents and young adults with anorexia nervosa

**DOI:** 10.1186/s40337-026-01612-y

**Published:** 2026-04-21

**Authors:** Anoushka Sinha, Rebecca K. Tsevat, May Lin, Amanda E. Downey, Sara M. Buckelew

**Affiliations:** 1 Department of Pediatrics, University of California, San Francisco (UCSF), 550 16th St, Box 0110, 94158 San Francisco, CA USA; 2 Department of Medicine, David Geffen School of Medicine, University of California, Los Angeles (UCLA), Los Angeles, CA USA; 3 Department of Neurology, University of California, San Francisco (UCSF), San Francisco, CA USA; 4 Department of Psychiatry and Behavioral Sciences, University of California, San Francisco (UCSF), San Francisco, CA USA

**Keywords:** Narrative medicine, Narrative-based intervention, Adolescent, Young adult, Anorexia nervosa, Eating disorder

## Abstract

**Purpose:**

Anorexia nervosa (AN) is highly morbid, particularly in adolescence. Narrative-based interventions, which employ storytelling and self-reflection, have improved outcomes for patients with various conditions, but narrative medicine specifically has not been studied in adolescents with AN. This study piloted and evaluated a narrative medicine clinical intervention for adolescents and young adults (AYAs) with AN in an outpatient setting.

**Methods:**

Participants (ages 16–25), stable for outpatient care and in therapy for AN, were recruited through eating disorder clinics and community referrals. The clinical intervention consisted of a 6-week narrative medicine workshop series around themes including identity, embodiment, and resilience. Surveys assessed the intervention’s acceptability, while clinical instruments assessed mental health and eating disorder symptoms; Wilcoxon signed-rank tests evaluated pre- and post-intervention differences. Semi-structured interviews elucidated participants’ experiences of the intervention, and thematic analysis identified emergent themes.

**Results:**

Eight participants enrolled, and seven completed the study. The mean age was 21 ± 4 years, and 75% were female. All participants found the intervention highly acceptable. Mean PHQ-9 scores improved after the intervention (13 to 10, *p* = 0.046). In qualitative analysis, four themes emerged: gaining insight into one’s condition, connecting over shared experiences, recognizing progress to be made, and viewing the workshops as a different type of treatment.

**Conclusions:**

This clinical intervention represents a novel application of narrative medicine for AYAs with AN. Narrative medicine was highly acceptable to participants, who derived meaning and social connection. Future studies may expand the intervention to larger, more diverse populations and further elucidate clinical outcomes and mechanisms for change.

**Trial registration:**

ClinicalTrials.gov (Identifier: NCT06849830).

**Supplementary Information:**

The online version contains supplementary material available at 10.1186/s40337-026-01612-y.

## Abstract (plain language summary)

Narrative-based interventions are structured practices that use listening and storytelling to improve outcomes for patients with various medical conditions. Narrative medicine, a type of narrative-based intervention, involves examining multimedia texts and practicing self-reflection in group settings. This is the first known study of narrative medicine for adolescents with anorexia nervosa (AN), an eating disorder associated with significant negative health outcomes. We recruited adolescents and young adults (AYAs) between the ages of 16 and 25 through clinics and community referrals. The intervention consisted of 6 weekly hour-long narrative medicine workshops around themes including identity, embodiment, and resilience. We measured impact by administering surveys about participants’ symptoms and conducting interviews about their experiences in the series. Eight participants enrolled, and seven completed the study. Narrative medicine was highly acceptable to all participants, led to a decrease in depression, and promoted social connection and meaning-making around illness. It will be important to expand this work to larger groups and determine how narrative-based interventions might complement existing forms of treatment for AYAs with AN.

**Supplementary Information:**

The online version contains supplementary material available at 10.1186/s40337-026-01612-y.

## Introduction

Anorexia nervosa (AN) is among the most lethal psychiatric conditions, with standardized mortality ratios approximately six times higher than in the general population, and confers particularly high risk among adolescents and young adults (AYAs) compared to age-matched peers [1,2]. Adolescence is a critical period for recognition and intervention, as it is characterized by rapid psychological and physiological changes, as well as heightened pressures around body image and social networks [3,4]. However, relapse is common even with medical stabilization and evidence-based treatments that address adolescent development, such as family-based treatment (FBT), and a significant proportion of patients do not fully weight restore or achieve full remission [5,6].

Given these high rates of relapse, it is critical to develop and evaluate novel clinical interventions that support sustained recovery. Narrative-based interventions, which engage individuals in storytelling, meaning-making, and self-reflection, have been effective in improving health outcomes for adults across a range of medical conditions, including symptom burden in patients with cancer and inflammatory bowel disease and glycemic control in patients with type 2 diabetes [8–10]. Additionally, narrative-based approaches have improved patient-centered outcomes such as well-being and quality of life in individuals with various types of illness [11,12]. Narrative medicine is one type of narrative-based intervention that integrates close reading of multimedia texts (including but not limited to poetry, visual art, film, and music) with reflective writing or drawing to foster reflection, storytelling, and meaning-making around illness [13]. One recent study examining a narrative medicine intervention for adults with AN demonstrated improvements in meaning-making and strengthened participants’ orientation toward recovery [14]. Despite these findings, narrative medicine remains largely unexplored in adolescent eating disorder treatment, and there are no known studies that evaluate associated health outcomes in this population.

Our pilot study aims to address this gap in adolescent eating disorder treatment by (1) introducing a novel narrative medicine intervention for AYAs with AN in an outpatient setting and (2) assessing its acceptability and preliminary impact on various clinical measures. Findings from this study may inform future adaptation and incorporation of narrative-based interventions into clinical care for AYAs with eating disorders.

## Methods

### Participants

Participants were recruited through flyers distributed at the University of California, San Francisco (UCSF) Eating Disorders Clinic and by referrals from local eating disorder providers, who were contacted via a community listserv. Medical stability and eligibility were assessed through intake screening and medical chart review by two co-investigators. Inclusion criteria required participants to be AYAs aged 16–25, diagnosed by a medical professional with AN, medically stable for outpatient care according to the Society of Adolescent Health and Medicine Clinical Guidelines [15], and actively engaged in outpatient psychotherapy for any duration of time by self-report. Exclusion criteria included active suicidal ideation or non-suicidal self-injury within the past two months, and/or a co-occurring personality disorder diagnosis. Participants received gift cards as compensation for their time and effort in the study. All procedures were approved by the UCSF Institutional Review Board. The study was registered on ClinicalTrials.gov (NCT06849830) prior to enrollment.

### Intervention

The intervention consisted of a six-week narrative medicine workshop series, held weekly from April to May 2025. Workshops were facilitated by two physicians trained in pediatrics, internal medicine, and/or AYA medicine, as well as narrative medicine. Each session was designed to reflect the established principles of narrative medicine, including action toward social justice, inclusivity, tolerance of ambiguity, and participatory and non-hierarchical methods [13].

Each one-hour workshop followed a consistent structure: an opening icebreaker (e.g., brief discussion of personal interests), close examination of multimedia texts (e.g., poetry, film, music, and visual art), a creative exercise (writing or drawing) based on a themed prompt, and voluntary group sharing and discussion. The workshops touched on issues related to eating disorders but were not explicitly focused on that aspect of participants’ experiences. Workshop themes included Connection, Identity, Communication, Embodiment, Resilience, and Hope. In planning and implementing the intervention, adjustments were continually made to the setting, delivery, and types of media explored based on participant feedback. For additional information about the intervention, see Appendix 1.

### Outcomes

Validated clinical measures included the Eating Disorder Examination Questionnaire (EDE-Q) to assess eating disorder symptoms, the Erikson Identity Subscale (EIS) to assess identity clarity, the Social Connectedness Scale (SCS) to measure sense of belonging, the Patient Health Questionnaire-9 items (PHQ-9) to evaluate depression, and the Generalized Anxiety Disorder-7 items (GAD-7) to evaluate anxiety. Clinical data, specifically percent of median body mass index (%mBMI), were extracted from participants’ electronic medical records. Changes in %mBMI were tracked for up to three months following the conclusion of the workshop series.

Acceptability of the intervention was measured by anonymous surveys administered after each workshop and at the conclusion of the series. These surveys included questions about participants’ enjoyment, learning, comfort, and perceived appropriateness of facilitation and content in the form of Likert scales (1–5).

To explore mechanisms of change and deepen understanding of the intervention’s impact, semi-structured interviews were conducted post-intervention by the study coordinator to assess participants’ experiences, perceived benefits, barriers to engagement, and interest in facilitating future narrative medicine groups. The semi-structured interview guide can be found in Appendix 2.

### Analysis

Descriptive statistics summarized demographic and clinical characteristics, as well as survey responses. Wilcoxon-signed rank tests evaluated pre- to post-intervention differences in clinical scales and %mBMI. For qualitative data, audio files were transcribed and reviewed for accuracy prior to analysis. Qualitative analysis was performed simultaneously using Dedoose software and followed a multi-step, iterative thematic analysis approach [16] Specifically, three members of the study team simultaneously coded each interview and identified emergent themes and sub-themes; discrepancies were resolved through consensus discussions to enhance rigor and reliability. Due to the exploratory nature of this pilot study, thematic sufficiency rather than saturation was the intended outcome. Sufficiency was determined when no substantively different themes emerged during analysis and was assessed independently by the two co-investigators throughout the analytic process. Because this was a preliminary pilot study, findings were intended to inform a larger subsequent trial. Of note, the research team included physicians with significant experience in the treatment of youth with eating disorders. Team members reflexively examined their own perspectives and biases related to the study content.

## Results

Eleven participants initiated contact with the study team and completed an intake interview to assess eligibility. Because recruitment occurred via flyers and listserv advertisements, it was not possible to determine how many individuals were exposed to the study invitation or approached overall. Of the eleven individuals who completed the intake, three did not enroll: one cited geographic barriers to participation, one withdrew due to competing personal obligations, and one was concurrently enrolled in a study with exclusionary criteria precluding simultaneous participation. Eight participants enrolled in the study, and seven completed the intervention. One participant dropped out of the study due to the need for inpatient hospitalization and subsequent residential treatment for worsening malnutrition. The mean age of enrolled participants (*n* = 8) was 21 *±* 4 years, and most identified as female (*n* = 6, 75%); the remainder (*n* = 2, 25%) identified as non-binary. Half of participants (*n* = 4, 50%) identified as White non-Hispanic; the remainder identified as Hispanic (*n* = 1, 13%), Black (*n* = 1, 13%), or Asian (*n* = 2, 25%). Their mean baseline %mBMI was 79 *±* 6.

In the post-series survey, participants largely found the intervention to be highly acceptable and felt comfortable participating in all parts of the intervention. All participants strongly agreed that the intervention was enjoyable, and they agreed that the intervention was educational, relevant to their interests and experiences, and appropriately structured and facilitated (Table 1).

**Table 1:** Post-intervention survey results (*n* = 7)

Survey item	Mean	SD
The series was enjoyable.	5	0
The content was relevant to my interests.	4.6	0.5
The content was relevant to my experience.	4.4	0.8
I learned something new as a result of this series.	4.4	1
The workshop format was appropriate.	4.7	0.5
The workshop facilitation was appropriate.	4.9	0.4
I felt comfortable participating in discussions.	4.1	0.9
I felt comfortable participating in reflective writing/drawing.	4.7	0.5

Scale: 1 = Strongly disagree, 2 = Somewhat disagree, 3 = Neither agree nor disagree, 4 = Somewhat agree, 5 = Strongly agree

The majority of participants also expressed interest in co-facilitating and/or advising future iterations of the workshops.

With regard to clinical measures, there was a signal toward improvement in mean PHQ-9 scores from baseline to post-intervention (13.3 to 10.3, *p* = 0.046); no changes were observed in GAD-7, EDE-Q, or SCS scores. In addition, %mBMI did not change from the beginning to the end of the intervention (Table 2).

**Table 2:** Clinical instruments (*n* = 7)

	Pre-Intervention Mean/SD	Post-Intervention Mean/SD	*P*-Value*
Patient Health Questionnaire-9	13.3 ± 5.5	10.3 ± 6.4	0.046
Generalized Anxiety Disorder-7	14.0 ± 6.2	12.3 ± 7.0	0.136
Social Connectedness Scale	78.8 ± 7.9	73.4 ± 11.0	0.223
Erikson Identity Subscale	37.5 ± 2.3	37.2 ± 4.6	0.672
Eating Disorder Examination Questionnaire	20.3 ± 9.7	17.7 ± 10.3	0.246
Percent Median Body Mass Index	79.1 ± 5.8	81.3 ± 4.6	0.116

*Obtained using the Wilcoxon Signed Rank Test

While thematic saturation was not an explicit goal of this exploratory study, we found that seven participant interviews yielded thematic sufficiency, with recurring patterns and rich accounts that allowed us to meaningfully address the research questions. Four themes emerged from the analysis: gaining insight into one’s condition, connecting over shared experiences, recognizing progress to be made, and viewing the workshops as a different type of treatment (Table 3).

**Table 3:** Emergent themes

Theme	Representative quotes
Gaining insight into one’s condition	“I feel like without the anorexia, I don’t know what I am, let alone who I am.” “It made me think more about my mental health, how…imagery or…paintings could reflect that.” “It definitely brought up some things I had forgotten about in the past, and I guess it made me more hyperaware of my eating disorder, and it reminded me that I shouldn’t ignore it. I should definitely understand my body more and…my emotional and physical needs more, and I shouldn’t really ignore them.” “I did notice a theme of…a person’s more likely to develop an eating disorder when they have something that…threatens their identity or sense of purpose or belonging in the world.”
Connecting over shared experiences	“Even if the group is only 7 people, those 7 people might as well have been the world population in terms of this experience.” “But…the real work just came in…connecting and reflecting with…another human.” “I think it was helpful being in a space where everyone’s been though similar things…it’s like everyone’s kind of doing it for each other at some point.” “My eating disorder has always been really personal, but…it made me more aware that other people, too, struggle with it, because at first…I just felt very in my own world about it.”
Recognizing progress to be made toward recovery	“I’m not even sure if I’m recovering, because…I feel like I want to get stronger but also don’t want it to impact my weight…I think I’m working on it.” “I mean, seeing numbers trend in a good direction, even slightly. My eating disorder freaks out over that. But health-wise, that means it’s a good thing.” “I really do want…a group setting for…recovery…I’m in therapy once a week and have been for many years, but…I’m really committed to my recovery this time around…this is the last time I want to recover from an eating disorder.” “As I nourish myself more, I’m able to come to the awareness of ways that I’m not showing up for other people, because I have…centered my eating disorder for so long.”
Viewing the workshops as a different type of treatment	“The experience was definitely new to me. I hadn’t done anything like that in the past where I would…share a space with others that…are also going through something similar.” “It was really cool to…meet people that I probably never would have met in any other capacity, even though we share so many identities.” “I’ve been to recovery programs where there’s just…group time. But it [was] never something quite like this.” “I wanted to be there versus not wanting to be at a different group therapy setting.”

### Theme 1: Gaining insight into one’s condition

Participants described “making sense of [their] experiences” through the intervention. Engaging in close reading and reflective writing with others allowed participants to become more vulnerable and “put that wall down a little bit.” This vulnerability enabled some participants to recognize their eating disorder as an illness; one described the intervention as a space where she “was the most raw [she] had been and confrontational directly about the eating disorder.” As she noted, “I feel like you have to name it to tame it.”

In addition to solely recognizing AN as an illness, participants came to acknowledge the deleterious impacts of their disorder—in some cases, considering it “a maladaptive coping skill.” One participant stated that engaging with the workshops “just shows me how much it’s not worth it to go through anorexia. It’s just…gonna take, even though it gives you the illusion of giving something.”

As they progressed through the intervention, participants not only acknowledged their illness but also sought ways to distance themselves from it. Through art, they were able to embrace other aspects of their identities and see themselves beyond their illness. One participant alluded to the “accessibility of art” for “when you feel so unseen and so vulnerable and insecure.” This accessibility, in turn, allowed participants to be less “self-critical,” and instead embrace “open-mindedness because art in itself is very open-minded.” Their open-mindedness allowed them to recognize and “[return] to the other outlets of…identity that aren’t revolving around the eating disorder,” noting that this frameshift “could be very healing.”

### Theme 2: Connecting over shared experiences

Participants reported that the reflections shared by group members enabled connection with others and reduced feelings of isolation related to their illness. Through participating in the intervention, one participant described feeling less alone because others “were going through similar things and had similar things to say.” Participants developed connections that took different forms. Some developed social bonds from hearing someone “put into words exactly how [they’re] feeling,” which was “deeply moving.” Others felt a “cognitive level of connection…not…a friend level of connection.” Additionally, they noted how these connections could facilitate self-understanding; as one stated, “it was nice to hear everyone else’s experiences in a non-triggering way…It made me understand mine.”

Participants described finding common ground and community through shared reflection about their illness. Some noted that they did not previously know anyone with the same diagnosis, stating, “I’ve never sat next to many people who were literally going through the exact same disorder I was going through, and talking to those people about it in real time made it more…real and more easily digestible.”

They also appreciated the points of contrast between their experiences of AN and the experiences of other group members, recognizing how “the idiosyncrasies of everyone just…shows…how complex this eating disorder is, but at the same time how universal our pain and journey is, too.” Hearing multiple views, some of which were different from their own, helped to shift their own perspectives. As one participant noted about her experience in the workshops, “there were many times where…someone would point out something that I didn’t even see…and it…has taught me to look at life in a different lens, too.”

### Theme 3: Recognizing progress to be made towards recovery

One of the shared experiences that participants named was the concept of recovery, and they noted that it was especially helpful to be “around people who are also committed to their recovery.” Seeing others in recovery was encouraging to some and allowed others to acknowledge their own recovery journeys. “I didn’t think I was recovering,” one participant said, recognizing the impact of the intervention. “So I mean, I guess it helped me understand that I am working towards it.”

Through the intervention, several participants came to define recovery not as a static state but rather a dynamic process. As one stated, “I’m at this point in recovery, where… I want to not only repair what has been damaged but grow new things in the aftermath of what I’ve learned, because… I know I’m not recovered, but… once I am fully recovered, I just want to live life.” This recovery process was not always linear and could include “many different ups and downs.” Participating in the intervention helped some to make “progress in terms of recovery.” While this progress was met with positive feelings for some, others felt more conflicted: as one noted, “I’m in a tug of war, and… I feel like I’m having more moments where I’m…slightly tugged in the direction of recovery… than [at] the beginning of the workshop.” Another participant expressed cynicism about the concept of recovery, reflecting that “there’s kind of a tinge of… oh…you’re in recovery again, you know. Like, sure, we’ll see if this time sticks.”

### Theme 4: Workshops as a different type of treatment

Finally, participants noted the ways in which this intervention differed from prior treatment modalities. Some described how the structure and form of the workshops were particularly conducive to self-reflection and creative expression; as one participant stated, “I think…writing about it through poetry, something that I enjoy…helped me process in a way that wasn’t so difficult. It was like an artistic lubricant.” Another participant explained how engaging in close reading and reflective writing was more productive than “[going] around and [sharing] all of our experiences related to anorexia,” which is commonly done in other treatment settings.

Others described how the experience of the intervention felt different from other therapeutic environments, which was primarily due to the characteristics of their co-participants. One said that the intervention was more “uplifting” than others because “in the eating disorder recovery space, it can be really easy to be…bogged down by people who are actively trying to get sicker or…competing with each other.” In contrast, they described fellow participants as “more open and honest in their responses, whereas…in a treatment center, no one really wants to be there, so it kind of feels like no one is really…super genuine.” While they did differentiate the intervention from other treatments, they also described how they could integrate these various modalities. One participant said she would debrief and process each workshop with her therapeutic team, noting that “my therapist…and…recovery coach [are] both…really glad that I went through with it. And we definitely…had some really good insights and conversation.” Others described the intervention as a gateway and an encouragement to explore other types of therapeutic group treatments.

## Discussion

This intervention represents a novel application of narrative medicine for AYAs with AN. Narrative medicine was highly acceptable to participants, who derived meaning and connection through the series. All participants found the intervention to be enjoyable, educational, relevant, and comfortable. In this exploratory analysis, there was also a signal of potential benefit in symptoms of depression, though these findings must be interpreted with caution as they are preliminary and unadjusted for confounders. Qualitative findings further illustrated that participants gained insight into their condition, connected with peers through shared reflection, and recognized progress to be made toward recovery. The cognitive and interpersonal experiences made possible by storytelling and reflective practice may represent mechanisms by which this narrative-based intervention contributed to improved mood symptoms.

Adolescence is a time of significant transition and is marked by an evolving understanding of self and others [17]. According to Erikson’s Stages of Development, adolescence and young adulthood are phases typically characterized by developing a coherent sense of identity and forming meaningful relationships [18]. When these processes go awry, individuals can struggle to grasp who they are and where they belong. These disruptions often occur among individuals with eating disorders, who can develop a rigid illness identity, feel isolated from peers and family, and struggle to see a future beyond their disorder [19]. Our participants described challenges with identity and isolation throughout the study but also discussed feeling empowered to overcome them through creative practice. These findings suggest that narrative medicine interventions can potentially tackle these issues by engaging with storytelling to help AYAs rebuild a sense of self and nurture meaningful connection.

An explicit focus on identity and social connection is often underemphasized by standard treatment models for eating disorders, which tend to prioritize the management of disordered cognitions and behaviors [5–7]. Conversely, by approaching the eating disorder obliquely through art and literature, participants identified the narrative medicine intervention as a space for self-exploration beyond the illness and an opportunity to recognize their strengths. In addition, participants appreciated having creative license to decide how much attention to devote to the eating disorder, which gave them agency to approach healing on their own terms and at their own pace. This control applied not only to what they chose to share but also to crafting the intervention itself, as the curriculum continually evolved in response to their feedback.

Other studies have reflected benefits of narrative-based interventions across a range of patient-centered outcomes. Specifically, studies of other AYA populations have demonstrated gains in reflective capacity, social connection, and creativity after participating in narrative-based interventions [20–22]. Although there are no known studies of narrative medicine for adolescents with AN, our qualitative findings were concordant with a pilot study of adults with AN, which showed improvements in identity clarity, social connection, and orientation toward recovery [14]. Because that study was limited to adults, the current findings are particularly salient; given that adolescence is a critical developmental period when individuals are striving to construct a sense of self and especially susceptible to social influences, patients of this age group may derive even greater benefit from narrative-based approaches as part of the recovery process [17,18]. Our study is also novel in exploring clinical outcomes relevant to eating disorders such as %mBMI, depression, anxiety, and eating disorder severity.

Limitations of this study include a small sample size, which had insufficient power to detect meaningful differences in quantitative findings. Moreover, the absence of a control group precludes causal inference regarding clinical improvements. Despite these constraints, the strong participation and retention rates, as well as rich qualitative data, provide encouraging preliminary evidence for perceived value in this population. Additionally, the duration of the study limits conclusions about longer-term effects. The study population was also a homogeneous sample from a single academic medical center, which limits generalizability. Selection bias likely influenced who chose to participate, and social desirability bias may have affected responses to surveys and interviews. To mitigate the latter concern, participants answered post-session surveys anonymously, and interviews were conducted by a research coordinator who did not lead the workshops.

Future studies with larger and more diverse cohorts will be necessary to evaluate clinical outcomes and mechanisms of action with greater precision. It will be important to determine whether hybrid or virtual formats, compared to in-person, are acceptable and efficacious for this population, given accessibility considerations. Finally, studies that employ a more participatory approach to intervention design are needed to ensure alignment with adolescents’ lived experiences, sustainability, and optimal impact.

## Conclusions

This pilot study offers preliminary evidence that narrative medicine may be a useful adjunct to traditional forms of treatment for young people with AN by supporting identity exploration and relational connection. These findings suggest that narrative practices may support progress toward recovery, decrease incidence of relapse, reduce feelings of isolation and depression, and re-engage aspects of identity that are often overshadowed by the illness. Expanding on this work will be essential for integrating narrative-based interventions into adolescent eating disorder care and for identifying the elements that most effectively complement existing therapeutic approaches.

## Supplementary Information

Below is the link to the electronic supplementary material.

Supplementary Material 1.

Supplementary Material 2.

## Data Availability

De-identified data are available upon reasonable request from the corresponding author.

## Abbreviations

AN: Anorexia nervosa
AYA: Adolescents and young adults
UCSF: University of California, San Francisco
EDE-Q: Eating Disorders Examination Questionnaire
EIS: Erikson Identity Subscale
SCS: Social Connectedness Scale
PHQ-9: Patient Health Questionnaire-9 items
GAD-7: Generalized Anxiety Disorder-7 items
%mBMI: Percent of median body mass index
