# Supplementary material for: “Name it to tame it”: piloting a narrative medicine clinical intervention for adolescents and young adults with anorexia nervosa
Source: J Eat Disord. 2026 Apr 21;14:126. doi: 10.1186/s40337-026-01612-y (PMC13237988; doi:10.1186/s40337-026-01612-y)
Supplement: Supplementary file 1 — Supplementary Material 1. [file 40337_2026_1612_MOESM1_ESM.docx]

**Appendix 2: Post-Series Interview Guide**

**Introduction:**

Thank you for meeting with me. Now that the narrative medicine series has ended, I would like to hear about your experience. There are no right or wrong answers—please feel free to share whatever comes to mind.

**1. Overall Impressions**

1. How would you describe your experience of the series?
2. What aspects of the experience felt meaningful, helpful, or positive?
3. What aspects, if any, felt less helpful or were more difficult to engage with?

**2. Identity Clarity**

1. In what ways, if any, has the series influenced how you think about yourself or your personal story?
2. Were there specific texts, prompts, or conversations that helped you reflect on your identity or story?

**3. Social Connectedness**

1. How would you describe your sense of connection with others in the group?
2. In what ways, if any, has your experience in the series influenced how you connect with people outside the group, such as family, friends, or members of your care team?

**4. Meaning-Making**

1. How did the series help you make sense of your experiences?
2. Were there moments or materials that felt especially meaningful or helped shift your perspective?

**5. Mental Health**

1. In what ways, if any, did the series affect your emotional or mental well-being?
2. Has your experience in the series influenced how you respond to challenges related to your health or other parts of your life?

**6. Overall Reflections and Suggestions**

1. What did you find most valuable about your experience in the series?
2. What suggestions would you offer for improving or adapting the series for future participants?

**7. Follow-up**

1. Would you be open to future contact to provide input on upcoming series? □ Yes □ No
2. Would you be interested in helping co-facilitate a future group? □ Yes □ No

a. *If not:* What might make you feel more open to that possibility?

**Conclusion:**

Thank you again for your time and reflections. Your feedback helps us better understand how participants experience the series and how it can continue to grow. Is there anything else you would like to share before we end?
