# Supplementary material for: “Name it to tame it”: piloting a narrative medicine clinical intervention for adolescents and young adults with anorexia nervosa
Source: J Eat Disord. 2026 Apr 21;14:126. doi: 10.1186/s40337-026-01612-y (PMC13237988; doi:10.1186/s40337-026-01612-y)
Supplement: Supplementary file 2 — Supplementary Material 2. [file 40337_2026_1612_MOESM2_ESM.docx]

**Appendix 1: Overview of Narrative Medicine Intervention**

| Session Theme | Description |
| --- | --- |
| Session 1:  Connection | Icebreaker: Tell us your happy place.  Texts: “Small Kindnesses” by Danusha Laméris (poem); scene from *Paterson* (film)  Prompts: Draw or write about a small kindness, or a time someone saw something in you that you didn’t recognize.  Take-home assignment: Bring or create something that aligns with your identity. |
| Session 2:  Identity | Icebreaker: Share the object you brought and how it aligns with your identity.  Texts: “Self Portrait” by Everlyn Nicodemus (painting); excerpt from *Stay True* by Hua Hsu (memoir)  Prompts: Draw a self-portrait, write about a time you did or did not stay true to yourself, or describe a portal to somewhere new. |
| Session 3: Communication | Icebreaker: Pick a question to answer (participants were given a bag from which they could pick out one of several questions).  Texts: “Communication” by Johnson Simon (painting); “Bird Understander” by Craig Arnold (poem)  Prompts: Draw or write about a failure of communication, or a time when language felt useless. |
| Session 4:  Embodiment | Icebreaker: Tell us the story of your name.  Texts: *Roots* by Frida Kahlo (painting); “What Am I Afraid Of?” by Sasha Debevec-McKenney (poem)  Prompt: Draw or write about your roots, or about what you are afraid of. |
| Session 5:  Resilience | Icebreaker: Tell us about a song that brings you joy or gives you strength.  Texts: “Wonder Woman” by Ada Limón (poem); “Everybody Hurts” by R.E.M. (song)  Prompts: Draw your superhero, write about the myth you need, or write about what “holding on” looks like for you.  Take-home assignment: Write a letter of hope to pass onto someone else in the group. |
| Session 6:  Hope | Icebreaker: Exchange your letter of hope with someone else, and read the letter you received aloud.  Texts: “The Letter” by Linda Gregg (poem); *The Song of the Lark* by Jules Breton (painting)  Prompts: Draw or write about what you hope for, or what lies on the horizon. |
